# Supplementary material for: MetaRibo-Seq measures translation in microbiomes
Source: Nat Commun. 2020 Jun 29;11:3268. doi: 10.1038/s41467-020-17081-z (PMC7324362; doi:10.1038/s41467-020-17081-z)
Supplement: Supplementary file 10 — Supplementary Data 7 [file 41467_2020_17081_MOESM10_ESM.zip › File2/Confidence_High_Taxonomy/195384_out.krona.html]

Javascript must be enabled to view this page.

members
magnitude
magnitudeUnassigned
count
unassigned
taxon
rank

195384\_out

3

3
2
superkingdom

phylum
1
1224

class
1
1236

135625
1
order

712
1
family

genus
1
724


SRS063373\_contig\_number\_contig-100\_6609.42565
1859695
1
species

phylum
1
1239

class
186801
1

186802
1
order

186804
1
family

1
1257

SRS142664\_contig\_number\_contig-100\_36806.36806
genus

976
1
phylum

1
200643
class

order
1
171549

171552
1
family

genus
838
1

species

SRS056327\_contig\_number\_11314
1
470565
